# Right-sided strangulating diaphragmatic hernia in an adult without history of trauma: a case report

**DOI:** 10.1186/s13256-021-02861-y

**Published:** 2021-07-13

**Authors:** Konstantinos G. Spiridakis, Mathaios E. Flamourakis, Ioannis G. Gkionis, Eleni I. Kaloeidi, Anthoula I. Fachouridi, Styliani E. Konstantoulaki, Eleni S. Tsagkataki, Michail I. Giakoumakis, Emmamouil A. Vassilogiannakis, Georgios E. Kostakis, Manousos S. Christodoulakis

**Affiliations:** 1 Department of General Surgery, Venizeleio General Hospital, Leoforos Knossou 44, Heraklion, Crete Greece; 2 Department of Imaging, Venizeleio General Hospital, Leoforos Knossou 44, Heraklion, Crete Greece

**Keywords:** Diaphragmatic hernia, Complications, Right-sided diaphragmatic hernia, Adult

## Abstract

**Background:**

Diaphragmatic hernia involves protrusion of abdominal contents into the thorax through a defect in the diaphragm. This defect can be caused either by developmental failure of the posterolateral foramina to fuse properly, or by traumatic injury of the diaphragm. Left-sided diaphragmatic hernias are more common (80–90%) because the right pleuroperitoneal canal closes earlier and the liver protects the right diaphragm. Diaphragmatic hernias in adults are relatively asymptomatic, but in some cases may lead to incarcerated bowel, intraabdominal organ dysfunction, or severe pulmonary disease. The aim of this report is to enlighten clinical doctors about this rare entity that can have fatal consequences for the patient.

**Case presentation:**

We present a rare case of a right-sided strangulating diaphragmatic hernia in an adult Caucasian patient without history of trauma. Clinical examination revealed bowel sounds in the right hemithorax, which were confirmed by the presence of loops of small intestine into the right part of the thorax through the right diaphragm, as was shown on chest X-ray and computerized tomography. Deterioration of the clinical status of the patient led to an operation, which revealed strangulated necrotic small bowel. Approximately 1 m of bowel was removed, and laterolateral anastomosis was performed. The patient had an uneventful postoperative recovery and was discharged 8 days later.

**Conclusions:**

Surgery is required to replace emerged organs into the abdomen and to repair diaphragmatic lesion. A delayed approach can have catastrophic complications for a patient.

## Introduction

Diaphragmatic hernia is a lesion of the diaphragm, through which loops of small and large bowel, stomach, liver, and spleen may protrude into the thoracic cavity of the involved side. This defect of the diaphragm can be either congenital or acquired, usually after a blunt trauma [1, 2]. Congenital diaphragmatic hernias are often classified by their position. A diaphragmatic hernia usually occurs in the posterolateral portion of the diaphragm (Bochdalek hernia), in 85% of cases [3]. In contrast, a Morgagni hernia is a defect involving the front part of the diaphragm, and this type accounts for approximately 2% of cases [4]. Left-sided diaphragmatic hernias are more common, because the right pleuroperitoneal canal closes earlier and the liver protects the right diaphragm [5].

In the vast majority of cases, diaphragmatic hernias are asymptomatic in adults [6]. In 5–10% of affected individuals, signs and symptoms of diaphragmatic hernia appear later in life and may include breathing problems or abdominal pain [7]. In some of these cases, a diaphragmatic hernia may lead to fatal complications such as strangulated intestine, intraabdominal organ dysfunction, or severe pulmonary disease [8, 9].

We report the case of a 50-year-old woman whose right-sided diaphragmatic hernia strangulated loops of small bowel and who was thus treated via urgent laparoscopy.

### Case presentation

We present the case of a 50-year-old Caucasian female patient who was hospitalized for vomiting and pain in the right upper abdomen and the right part of the thorax. She was ill-looking. Her vital signs on admission were temperature 36.8 °C, heart rate 70 beats per minute, respiratory rate 17 breaths per minute, and blood pressure 120/80 mmHg.

Her body mass index was 20, her tobacco use was 5 pack-years, and she did not consume alcohol. She was married and had two children aged 14 and 19 years. She was employed in a bank in Belgium, where she lived permanently. She was in vacation with her family in Greece at the time of her admission to the hospital.

She was taking no medication and had no other underlying disease. Furthermore, her medical history was unremarkable, without any previous surgical interventions in the abdomen or thorax. There was no history of any previous abdominal or thoracic trauma.

On clinical examination, the abdomen was not tender, even in the right upper abdomen, but the presence of bowel sounds in the right hemithorax was revealed through stethoscope. There were no findings on physical and neurological examination.

The blood tests depicted an elevation of inflammatory markers (white blood cell count 16,900/μL, normal range 3,800–10,500/μL; C-reactive protein 0.1, normal values < 0.05) and a slight deterioration of renal function (Ur 63, normal range 15–50; Cr 1.38, normal range 0.7–1.3). The results of all other markers were within normal range (hemoglobin 13.6, normal range 13.4–17.4; hematocrit 43, normal range 41.0–53.8; platelet count 250.000, normal range 150.000–400.000; serum glutamic-oxaloacetic transaminase 30, normal range 5–35; serum glutamic-pyruvic transaminase 31, normal range 0–55; γ-glutamyl transferase 48, normal range 0–50; sodium 137, normal range 136–145, potassium 4.5, normal range 3.5–5.1). Blood cultures were negative for bacterial growth.

**Fig. 1:**
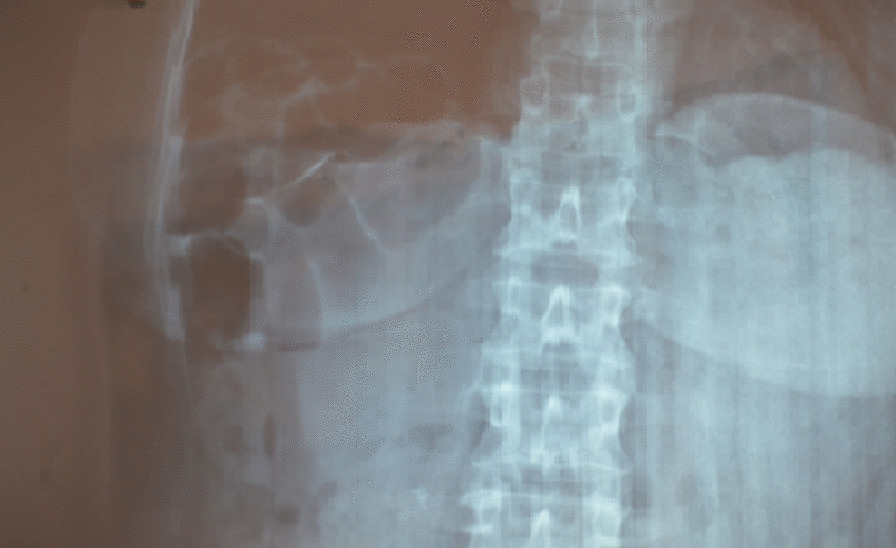
X-ray, loops of small bowel into the right part of the thorax

**Fig. 2:**
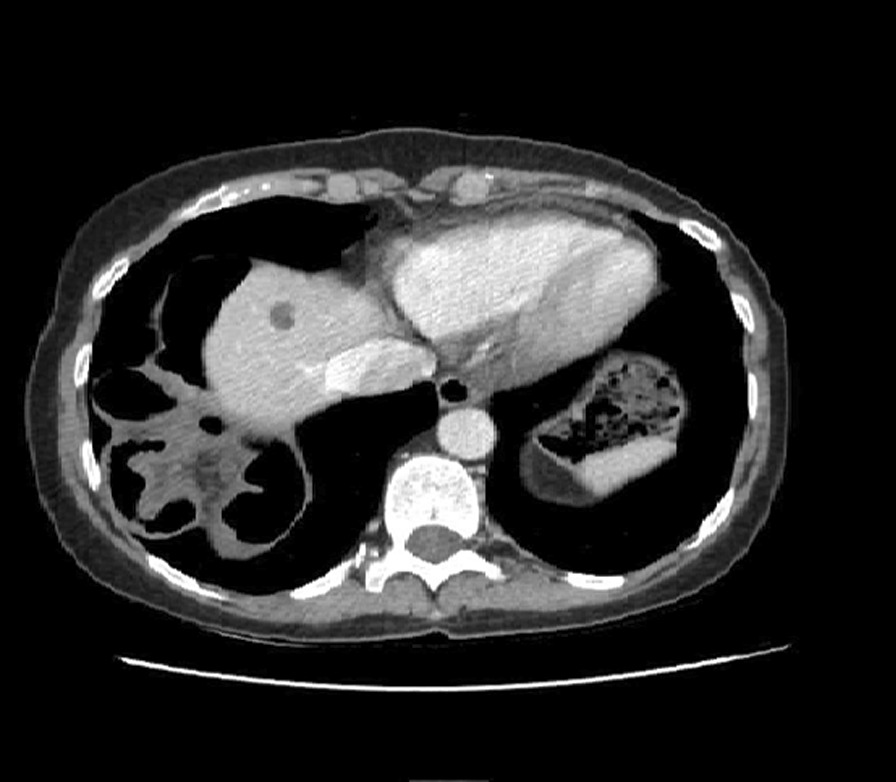
CT scan, right-sided diaphragmatic hernia

Chest X-ray revealed a right elevation of the diaphragm with the presence of small bowel into the right thoracic cavity (Fig. 1). Computerized tomography of the chest and abdomen was performed and confirmed the presence of loops of small intestine into the right hemithorax through the right diaphragm (Fig. 2).

Due to deterioration of her clinical status with increasing pain in the right upper abdomen and right hemithorax, the patient underwent urgent laparoscopy 12 hours after her admission to the hospital. The operation revealed strangulated necrotic small bowel inside the diaphragmatic hernia. The initial laparoscopy was converted into laparotomy. Due to the strangulation, a part of the small bowel was ischemic and necrotic. A total of 80 cm of small bowel and 10 cm of large bowel (cecal) was removed because the necrosis of the intestine was near the ileocecal valve. Laterolateral anastomosis was performed, and the 5-cm wide defect in the diaphragm was repaired with interrupted sutures. During the surgical procedure, a chest tube was inserted.

The postoperative period was without any incident, and the patient was released in good condition 8 days after the operation. After the surgery and during her hospital stay, she was receiving intravenous 3 g cefoxitin/day for 3 days, 1.5 g metronidazole/day for 3 days, and 4 g paracetamol/day for 4 days. The initial postoperative intravenous administration of fluids was followed by oral feeding after 4 days.

The patient lives permanently in Belgium, and after 3 months of the operation, she sent findings of computerized tomography in the chest and abdomen showing no pathological entity.

## Discussion

We report a rare case of a right-sided diaphragmatic hernia in a 50 year-old woman without previous history of trauma. Clinical examination and imaging of chest and abdomen revealed the presence of loops of small bowel into the right part of the thorax. Urgent laparoscopy revealed the reason for the deterioration of her clinical status, as part of the small bowel inside the hernia was ischemic and necrotic. Abdominal viscera into the thoracic cavity can be developed after injury to the chest or due to congenital defect of the diaphragm. Left-sided diaphragmatic hernias are more common, because the right pleuroperitoneal canal closes earlier and the liver protects the right diaphragm [5]. It is extremely rare to detect a right-sided diaphragmatic hernia in an adult without trauma [5]. Up to 2004, only ten such cases had been reported [10–12]. Since 2004, ten more cases have been added [13].

Most diaphragmatic hernias are diagnosed in children who present with acute pulmonary symptoms [5]. It occurs in about 1 in 2200–12,500 live births [5]. In contrast to the acute presentation in infants, diaphragmatic hernias in adults are relatively asymptomatic [6]. The absence of breath sounds and the presence of bowel sounds in the chest are typical findings associated with diaphragmatic hernia [6, 7].

In most of the cases, the disorder is unexpectedly detected on chest X-ray [1]. Due to the low sensitivity of chest radiography, diaphragmatic hernias may be confused for other thoracic pathologies, including tension pneumothorax, pericardial fat pad, sequestration of the lung, mediastinal lipoma, or anterior mediastinal mass [1, 14]. The gold standard technique for diagnosis is computerized tomography, which enables clinical doctors to evaluate the size, location, and type of diaphragmatic hernia [14]. Two studies revealed that computerized tomography has a sensitivity of 78% for left-sided hernias and 50% for right-sided hernias [1, 14].

The most common symptoms in affected adults include chronic dyspnea, chest pain, recurrent abdominal pain, postprandial fullness, and vomiting [6, 7]. The lack of specific clinical signs may delay the correct diagnosis, which can have fatal consequences for the patient. An undiagnosed diaphragmatic hernia may lead to strangulated intestine, intraabdominal organ dysfunction, or severe pulmonary disease with a mortality rate of 32% [8, 9].

Carter *et al.* described the four stages in the development of strangulating diaphragmatic hernia, and they believe that it is important for clinical doctors to recognize each of these four stages in the eventual development of a diaphragmatic hernia [15]: (1) asymptomatic, (2) minimal symptoms, (3) obstruction, and (4) strangulation. The stage of obstruction is characterized by severe upper abdominal or lower thoracic pain, nausea, and vomiting. In the fourth stage, the tension of the symptoms is increased, and a surgical intervention is necessary. Most of the patients develop the last complication (strangulation) weeks, months, or even years after the first diagnosis of a congenital or posttraumatic diaphragmatic hernia, as reported by Carter *et al*. [15]. In cases with diaphragmatic hernia, rapid diagnosis and eventual surgical intervention are important. A delayed approach can have catastrophic complications for a patient [8, 9].

In our case, the patient was hospitalized for vomiting and pain in the right upper abdomen and the right part of the thorax, which are the main symptoms depicted in literature for strangulating diaphragmatic hernia [6–9, 15]. Computerized tomography enabled us to evaluate the size, location, and type of diaphragmatic hernia. The elevation of the tension of the symptoms led without delay to a surgical intervention, which was lifesaving for the patient.

## Conclusion

We report a rare case of right-sided diaphragmatic hernia in an adult who was treated via urgent laparoscopy. Despite being rare, this disorder should be recognized, examined, and treated appropriately to avoid fatal complications. However, there is no consensus among surgeons regarding the timing and the absolute indications of a surgical intervention. Early diagnosis is very important for the urgent surgical intervention, which remains the only curative treatment for diaphragmatic hernia associated with strangulated abdominal organ.

## Data Availability

The datasets generated and analyzed during the current study are available from the corresponding author on reasonable request.
